# Terpenoids and Phenylpropanoids in *Ligularia duciformis*, *L. kongkalingensis*, *L. nelumbifolia*, and *L. limprichtii*

**DOI:** 10.3390/molecules22122062

**Published:** 2017-11-25

**Authors:** Chiaki Kuroda, Ryohei Kobayashi, Ayumi Nagata, Yumi Nakadozono, Taketo Itoh, Yasuko Okamoto, Motoo Tori, Ryo Hanai, Xun Gong

**Affiliations:** 1 Department of Chemistry, Rikkyo University, Nishi-Ikebukuro, Toshima-ku, Tokyo 171-8501, Japan; 13cc013b@rikkyo.ac.jp (R.K.); 12cc123d@rikkyo.ac.jp (A.N.); 13cc039h@rikkyo.ac.jp (Y.N.); 13cc128f@rikkyo.ac.jp (T.I.); 2 Faculty of Pharmaceutical Sciences, Tokushima Bunri University, Yamashiro-cho, Tokushima 770-8514, Japan; yasuko@ph.bunri-u.ac.jp (Y.O.); tori@ph.bunri-u.ac.jp (M.T.); 3 Department of Life Science, Rikkyo University, Nishi-Ikebukuro, Toshima-ku, Tokyo 171-8501, Japan; hanai@rikkyo.ac.jp; 4 Kunming Institute of Botany, Chinese Academy of Science, Kunming 650201, China; gongxun@mail.kib.ac.cn

**Keywords:** *Ligularia duciformis*, *Ligularia kongkalingensis*, *Ligularia nelumbifolia*, *Ligularia limprichtii*, eremophilane, oplopane, ITS sequence, diversity

## Abstract

The diversity in root chemicals and evolutionally neutral DNA regions in the complex of *Ligularia duciformis*, *L. kongkalingensis*, and *L. nelumbifolia* (the d/k/n complex) was studied using eight samples collected in central and northern Sichuan Province of China. Cacalol (**14**) and epicacalone (**15**), rearranged eremophilanes, were isolated from the complex for the first time. Two new phenylpropanoids were also obtained. Seven of the eight samples produced phenylpropanoids and the other produced lupeol alone. Two of the seven samples also produced furanoeremophilanes or their derivatives and one produced oplopanes. The geographical distribution of the sesquiterpene-producing populations suggests that the production of sesquiterpenes evolved independently in separate regions. *L. limprichtii* collected in northern Sichuan was also analyzed and its chemical composition and the sequence of internal transcribed spacers (ITSs) in the ribosomal RNA gene cluster were found to be similar to that in the d/k/n complex and *L. yunnanensis*, which are morphologically similar.

## 1. Introduction

Species belonging to the genus *Ligularia* are highly diversified in the Hengduan Mountains area of China and their evolution is considered to be continuing [1]. Most of major *Ligularia* species in the Hengduan Mountains area produce furanoeremophilanes as major root chemicals [2,3]. We previously proposed a hypothesis that furanoeremophilane-producing species or intra-specific groups are ecologically advantageous over those producing eremophilan-8-ones [2]. Eremophilan-8-ones are precursors of various furanoeremophilanes [4].

*Ligularia duciformis* (C. Winkl.) Hand.-Mazz., *L. kongkalingensis* Hand.-Mazz., and *L. nelumbifolia* (Bureau & Franch.) Hand.-Mazz. are abundant in the Hengduan Mountains area. The three species are morphologically close to one another and their differentiation is based on the pili on the involucres (pilose in *L. kongkaligensis* or glabrous in *L. duciformis* and *L. nelumbifolia*) and on the length of pappi (shorter in *L. duciformis*, longer in *L. nelumbifolia*, and intermediate in *L. kongkalingensis*) [5,6]. However, in our observation, morphological characters appear continuous. In addition, the three species are indistinguishable with respect to root chemicals and evolutionally neutral DNA [7], which have been used as the indices of diversity in our series of work on *Ligularia* [2,3]. Thus, the three species can be considered to form a complex, which we call the d/k/n complex in this report.

We previously analyzed 28 samples of the d/k/n complex and classified them into four chemotypes [7]: an eremophilane-producing type (type 1), an oplopane-producing type (type 2), a phenylpropanoid-producing type (type 3), and a type without sesquiterpenoid and phenylpropanoid (type 4). Most of the samples were of type 3. Phenylpropanoids were isolated also from types 1 and 2. Although no furanoeremophilane was detected in these 28 samples [7], a *L. nelumbifolia* sample, collected thereafter in Zhegushan, Hongyuan County in northern Sichuan Province, was found to produce furanoeremophilane [8]. This led us to conduct a further search in central and northern Sichuan during 2011–2015. Here we report the presence of another furanoeremophilane-producing individual as well as an individual producing cacalol, a rearranged furanoeremophilane.

A sample of *L. limprichtii* (Diels) Hand.-Mass. collected in 2010 in northern Sichuan was also analyzed. *L. limprichtii* is similar to the d/k/n complex in morphology. The major differences are in the involucres (narrowly cylindric to campanulate-cylindric in the d/k/n complex or campanulate-turbinate in *L. limprichtii*) and in the leaf blade (glabrous in the d/k/n complex or abaxially shortly pilose in *L. limprichtii*) [5]. To the best of our knowledge, its chemical composition has not been reported. Here we report the isolation of a phenylpropanoid from *L. limprichtii*.

## 2. Results

### 2.1. Samples

**Figure 1:**
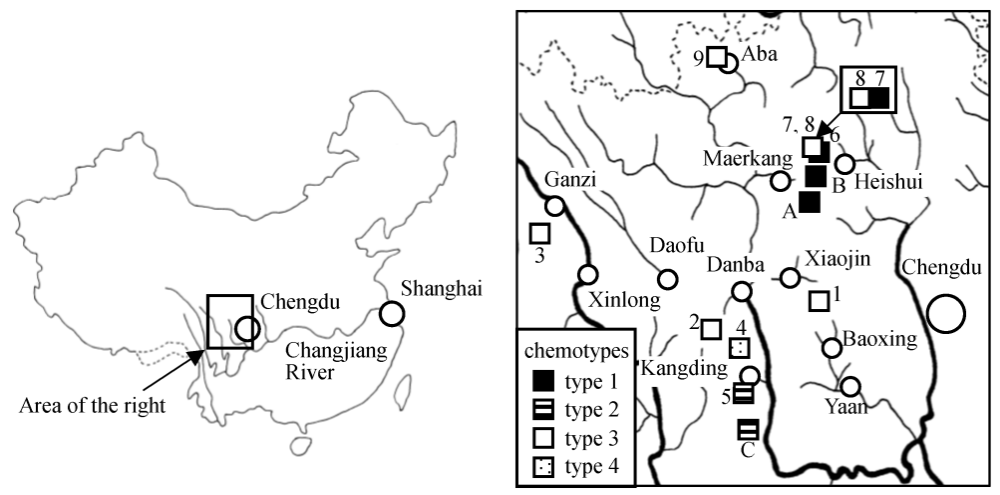
Locations of samples of *L. duciformis*, *L. kongkalingensis*, *L. nelumbifolia*, and *L. limprichtii*. Rectangles, samples; circles, cities; solid lines, rivers; dotted lines, boundaries of provinces. A–C are previously collected type-1 and type-2 samples within the map area [7,8].

Samples of the d/k/n complex (samples 1–8) and a sample of *L. limprichtii* (sample 9) were collected in the field shown in Table 1 and Figure 1. The locations of three previously collected sesquiterpene-producing samples are also included in Figure 1 as samples A, B, and C for reference.

### 2.2. Chemical Constituents

For a characterization of the chemical composition of the samples, an EtOH extract of the fresh root of each sample was analyzed by TLC using Ehrlich’s coloring reagent, a facile method to detect furanoeremophilanes [9,10]. Sample 7 showed Ehrlich-positive spots, the most prominent at *R*_f_ = 0.65 (hexane/EtOAc 7:3), suggesting the presence of furanoeremophilanes, although the spots were weaker than for typical furanoeremophilane-producing *Ligularia* plants. Sample 6 showed very weak spots, and samples 1–5, 8, and 9 were negative to the test.

**Figure 2:**
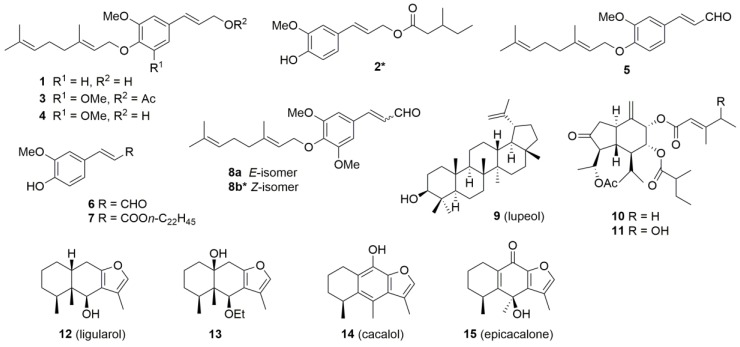
Isolated compounds from *L. duciformis*/*L. kongkalingensis*/*L. nelumbifolia* (samples 1–8) and *L. limprichtii* (sample 9). Asterisks indicate new compounds.

For the isolation of compounds, dried roots of each sample were extracted with EtOH, and the compounds therein were separated by silica-gel column chromatography and HPLC as usual. Fifteen compounds, **1**–**15**, were isolated from the eight samples (Table 1 and Figure 2). From samples 1–3, 8, and 9, phenylpropanoids were isolated without terpenoids. The isolated compounds were **1** [11] and **5** [7] from sample 1; **3** [12,13], **7** [14], and lupeol (**9**) [15] from sample 2; **3** and **4** [11,13,14] from sample 3; **3**, **4**, and **8** (a mixture of known compound **8a** [14]; and new compound **8b** at a ratio of 7:1) from sample 8; and **3** from sample 9. From sample 4, lupeol (**9**) alone was isolated as in the previously reported type-4 samples [7]. Sesquiterpenes were isolated from samples 5–7. From sample 5, oplopanes **10** [7] and **11** [16] were isolated in addition to a new compound **2**, coniferaldehyde (**6**) [17], and **9**. From sample 6, cacalol (**14**) [18,19] and epicacalone (**15**) [20] were isolated together with **3**, **4**, **8**, and **9**. From sample 7, two furanoeremophilanes, ligularol (**12**) [21] and **13** [22], were isolated.

**Figure 3:**
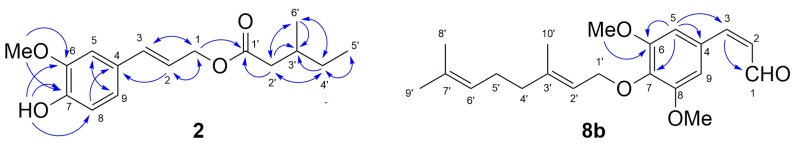
Selected HMBC correlations of compounds **2** and **8b**.

The structure of the new compound **2** was determined as follows. The molecular formula of **2** was determined to be C_16_H_22_O_4_ from the high-resolution mass spectrum (*m*/*z* 278.1510). The IR (1730 cm^−1^) and ^13^C-NMR spectra (δ 173.3) showed the presence of an ester carbonyl group. The ^1^H and ^13^C-NMR spectra showed typical signals of a coniferyl ester moiety, i.e., signals of a methoxy group [δ_H_ 3.91 (s); δ_C_ 55.9], an oxygenated methylene [δ_H_ 4.71 (dd, *J* = 6.6, 1.1 Hz); δ_C_ 65.0], an *E*-alkene [δ_H_ 6.14 (dt, *J* = 15.7, 6.6 Hz) and 6.58 (d, *J* = 15.7 Hz)], and a 1,2,4-trisubstituted aromatic ring [δ_H_ 6.87 (d, *J* = 7.6 Hz), 6.90 (dd, *J* = 7.6, 1.5 Hz), and 6.92 (d, *J* = 1.5 Hz)] (See Section 4.3 and Figures S1 and S2). Signals of two methyl groups were observed as a triplet (δ_H_ 0.90) and a doublet (δ_H_ 0.95) in addition to those of two methylenes and one methine (δ_H_ 1.91), the latter of which was observed in octet, suggesting that the acid part was 3-methylpentanoate. The structure was established from COSY observed for 2′-H/3′-H/4′-H/5′-H and 3′-H/6′-H and from HMBC correlations shown in Figure 3.

Compound **8** was obtained as an inseparable mixture of *E*- (**8a**) and *Z*-isomers (**8b**), the latter of which was new. Although a full characterization of **8b** was not feasible due to signal overlap with **8a**, its structure was deduced to be the *Z* isomer from its ^1^H-NMR spectrum, as the *J*-value between 2-H (δ_H_ 6.15) and 3-H (δ_H_ 7.54) was 11.4 Hz (See Section 4.4 and Figure S3). Although all ^1^H signals of the geranyl moiety of **8b** overlapped with those of **8a** except for 1′-H, the signals of the sinapylaldehyde part were separated from those of **8a**. The structure was confirmed by HMBC correlations shown in Figure 3 (See Section 4.4 and Figure S4 for the ^13^C-NMR spectrum).

**Figure 4:**
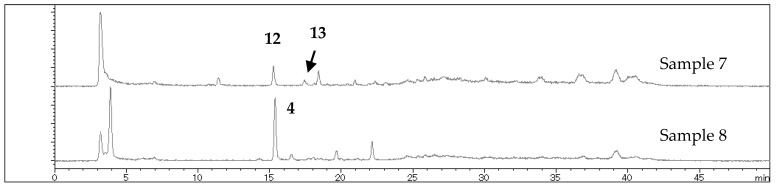
LCMS profile (total ion chromatogram) of samples 7 and 8. See Figure S5 for samples 1–6 and 9.

The chemical compositions of the d/k/n complex samples were compared by LC-MS analysis. Samples 1–6 and 9 showed no distinct peaks in *t*_R_ = 10 to 30 min (MeOH/H_2_O, see Section 4.1 for the experimental conditions) where typical terpenoids and phenylpropanoids were expected (Figure S5). Large signals were observed in *t*_R_ = 3 to 4 min, however, we could not identify the compounds. In contrast, ligularol (**12**) and 6β-ethoxyfuranoeremophilan-10β-ol (**13**) were detected at *t*_R_ = 15.3 and 17.5 min, respectively, in the chromatogram of sample 7 (Figure 4). Compound **13** may be an artifact generated during EtOH extraction [22], and its presumed parent compound, furanoeremophilane-6β,10β-diol, is also detected at *t*_R_ = 11.5 min. Sample 8 showed one major peak of *O*-geranylsinapyi alcohol (**4**) at *t*_R_ = 15.4 min, which was isolated as the major component (Figure 4). 

### 2.3. Genetic Study

To assess the genetic diversity among the samples, the DNA sequence was determined for the internal transcribed spacer 1 (ITS1)-5.8S-ITS2 region of the ribosomal RNA gene. The results are shown in Table S1. Although there are some variations, a bootstrapping analysis by the UPGMA method using MEGA6 [23] found no distinct clade among the samples. The result indicates that *L. limprichtii* is genetically similar to the d/k/n complex.

## 3. Discussion

### 3.1. Chemotypes

We previously grouped d/k/n complex samples, collected in western Sichuan and northwestern Yunnan, into four chemotypes: type 1 (eremophilanes), type 2 (oplopanes), type 3 (phenylpropanoids), and type 4 (no phenylpropanoid nor sesquiterpenoid) [7,8]. According to this classification, sample 7 belongs to type 1; sample 5, type 2; samples 1–3, and 8, type 3; and sample 4, type 4 (Table 1). Cacalol (**14**) and epicacalone (**15**) have a rearranged carbon skeleton generated from furanoeremophilane [18], and thus, sample 6 also belongs to type 1. While cacalol is a compound often found in *Ligularia* species, such as *L. virgaurea* [24] and *L. cyathiceps* [25], sample 6 is the first example of the isolation of cacalol from the d/k/n complex.

Two of our previous type-1 samples were collected in Maerkang, Sichuan (samples A and B in Figure 1) [7,8]. With the present two additions, samples 6 and 7, it can be said that type 1 is abundant in the Maerkang-Heishui area. It is interesting that this area harbors all the three evolutionary stages of eremophilane biosynthesis: non-furano-eremophilane, furanoeremophilane, and cacalol. Sample 5 is the fourth example of type 2 (oplopane). Phenylpropanoids were also obtained from the present type-1 and type-2 samples as observed previously [7]. In particular, the major component in sample 6 was *O*-geranylsinapyl alcohol (**4**), a phenylpropanoid typical of type 3.

Samples 1–3 and 8 belong to type 3. While *O*-geranylsinapyl alcohol (**4**) was the major component in sample 8 (8.3% of the extract), phenylpropanoids in samples 1–3 were small in quantity. Although lupeol (**9**) was the sole compound isolated from sample 4, the possibility cannot be excluded that it contained a very small quantity of phenylpropanoids. More than half of the type-3 samples in our previous study produced lupeol (**9**) as well as phenylpropanoids [7]. Thus, type 3 and type 4 may constitute one chemotype with a continuous composition spectrum.

### 3.2. Introgression and Chemical Evolution

We previously proposed that introgression might have brought the ability to produce sesquiterpenes from some other plant into type 3, resulting in types 1 and 2 [7]. This premise has found support in the discovery of hybrids between *L. duciformis* and *L. cyathiceps* [26] and hybrids between *L. nelumbifolia* and *L. subspicata* [27] and the isolation of furanoeremophilanes, the major components of both *L. subspicata* and *L. cyathiceps*, from them. Although the present DNA data did not separate samples 6 and 7 from the other samples, the few sites of multiple bases, particularly in sample 7, may be suggestive of such introgression (Table S1). Type 1 has been found in Shangrila, Yunnan, and Maerkang, Sichuan. Since these areas are distant from each other, the evolution of eremophilane production is likely to have happened independently. The location of sample 5 (type 2) was not far (ca. 30 km) from one of the previous two type-2 samples (sample C in Figure 1) but far from the other in Shangrila, Yunnan. Their evolution is also likely to have been independent.

### 3.3. L. limprichrii

*O*-Geranylsinapyl acetate (**3**) was isolated from *L. limprichtii* (sample 9). The same compound was also obtained from samples 2 and 3, as well as eight of the 18 type-3 samples in our previous study [7]. Phenylpropanoids have been isolated from taxonomically related species, *L. yunnanensis* (Franch.) Chang [26] and *L. purdomii* (Trrill) Chittenden [28], from the latter of which eremophilanes and euparin-type benzofurans were also isolated. DNA data also show the similarity as the ITS1-5.8S-ITS2 sequence of sample 9 cannot be separated from those of other samples (Table S1). The sequence of *L. yunnanensis* is indistinguishable from that of *L. duciformis* [26], therefore, *L. limprichtii* is similar to both the d/k/n complex and *L. yunnanensis*. Further study with more samples is necessary to understand the relationship among the d/k/n complex, *L. limprichtii*, and other taxonomically related species.

## 4. Materials and Methods

### 4.1. General Experimantal Procedure

Specific rotations were measured on a DIP-370 polarimeter (JASCO, Tokyo, Japan). NMR spectra were measured on an ECX-400 or an AL-400 (400 MHz for ^1^H; 100 MHz for ^13^C) spectrometer (JEOL, Tokyo, Japan). IR spectra were measured on a FT/IR-230 spectrometer (JASCO), and MS spectra, on a JMS-700 MStation or a CMATE II (JEOL). Kieselgel 60 F254, 0.2 mm thickness (Merck, Darmstadt, Germany) was used for analytic TLC, with either Ehrlich’s reagent (*p*-dimethylaminobenzaldehyde and HCl) [9,10] or *p*-anisaldehyde/AcOH/H_2_SO_4_ as visualizing agents. Open column chromatography (CC) was carried out on silica gel (Wakogel C-200 (Wako, Kyoto, Japan) or silica gel 60 N (Kanto, Tokyo, Japan)). HPLC with a Mightysil Si60 (10 mm × 250 mm) column (Kanto) was carried out on either an LC-20AT pump with a SPD-20A Prominence UV/VIS detector (Shimadzu, Kyoto, Japan) or a GL-7410 pump with a GL-7450 UV detector (GL Sciences, Tokyo, Japan), and with a D-2500 Chromato-Integrator (Hitachi, Tokyo, Japan) or a C-R8A Chromatopac recorder (Shimadzu). LC-MS was measured on a 1100 series LC/MSD mass spectrometer (capillary voltage 3.5 kV; corona current 4 μA; capillary exit voltage (fragmentor) 90 V; drying temperature 330 °C; drying flow 9 L/min; nebulizer pressure 50 psig) (Agilent, Santa Clara, CA, USA) with 5C18-MS-II (COSMOSIL; 4.6 mm × 150 mm; 5 μm octadecyl column (Nakarai, Kyoto, Japan)) using gradient system (MeOH/H_2_O; 0 min (7:3)—20 min (10:0)—35 min (10:0)—40 min (7:3)—45 min (7:3); 0.5 mL/min) as eluent. DNA was purified from dried leaves using DNeasy Plant Mini Kit (QIAGEN, Hilden, Germany). Polymerase chain reaction was carried out using L5 and L6 primers previously described [29] and HotStarTaq plus Master Mix (QIAGEN). The amplification products were separated by agarose gel electrophoresis and purified with High Pure PCR Product Purification Kit (Roche Diagnostics, Basel, Switzerland). DNA sequencing reaction was carried out using L1–L4 primers described previously [29] and BigDye Terminator v3.1 Cycle Sequencing Kit (Applied Biosystems, Waltham, MA, USA) and analyzed on a 3130xl or 3500 Genetic Analyzer (Applied Biosystems).

### 4.2. Plant Materials

Samples were collected in August 2010, 2011, 2012, and 2015 at locations shown in Table 1 and Figure 1. Each sample was identified by X. G. (author). Voucher specimen numbers are 2011-12, 2011-29, 2011-82, 2012-10, 2012-28, 2015-33, 2015-37, 2015-40, and 2010-32, for samples 1–9, respectively (Kunming Institute of Botany, Kunming, China).

### 4.3. Extraction and Purification

The dried roots of sample 1 (43.0 g) were extracted with EtOH. The extract (1799.4 mg) was subjected to silica-gel (20 g) CC using *n*-hexane/EtOAc (gradient) as the eluent to obtain eight fractions. Fr. 3 (eluted with *n*-hexane/EtOAc 96:4) was further separated by CC (silica gel, *n*-hexane/EtOAc) to afford seven fractions (Fr. 1–1 to 1–7). From Fr. 1–5, **5** (0.3 mg) was obtained by HPLC (Mightysil, *n*-hexane/EtOAc 7:3). From Fr. 1–7, **1** (6.5 mg) was obtained by HPLC (*n*-hexane/EtOAc 7:3).

The dried roots of sample 2 (57.4 g) were extracted with EtOH. The extract (219.1 mg) was subjected to silica-gel (6 g) CC using *n*-hexane/EtOAc (gradient) as the eluent to obtain seven fractions. From Fr. 3 (eluted with *n*-hexane/EtOAc 9:1), **9** (1.6 mg) was isolated by repeated CC and HPLC (*n*-hexane/EtOAc 1:1). From Fr. 4 (eluted with *n*-hexane/EtOAc 8:2), **7** (3.1 mg) and **3** (1.0 mg) were obtained by CC and HPLC (*n*-hexane/EtOAc 7:3).

The dried roots of sample 3 (13.1 g) were extracted with EtOH. The extract (218.5 mg) was subjected to silica-gel (12 g) CC using *n*-hexane/EtOAc (gradient) as the eluent to obtain eight fractions. From Fr. 6 (eluted with *n*-hexane/EtOAc 9:1 to 8:2), **3** (1.1 mg) was obtained by CC and HPLC (*n*-hexane/EtOAc 8:2 to 3:1). From Fr. 8 (eluted with *n*-hexane/EtOAc 8:2), **4** (5.1 mg) was obtained by HPLC (*n*-hexane/EtOAc 1:1).

The dried roots of sample 4 (48.5 g) were extracted with EtOH. The extract (3060.7 mg) was subjected to silica-gel (12 g) CC using *n*-hexane/EtOAc (gradient) as the eluent to obtain 10 fractions. From Fr. 3 (eluted with *n*-hexane), **9** (1.8 mg) was isolated by repeated CC (silica gel, *n*-hexane/EtOAc) and HPLC (*n*-hexane/Et_2_O 6:4).

The dried roots of sample 5 (70.3 g) were extracted with EtOH. The extract (639.4 mg) was subjected to silica-gel (12 g) CC using *n*-hexane/EtOAc (gradient) as the eluent to obtain eight fractions. From Fr. 4 (eluted with *n*-hexane/EtOAc 9:1), **9** (20.1 mg) was isolated by CC and HPLC (*n*-hexane/EtOAc 7:3). From Fr. 5 (eluted with *n*-hexane/EtOAc 85:15), **2** (0.6 mg) and **10** (2.5 mg) were obtained by CC and HPLC (*n*-hexane/EtOAc 7:3). From Fr. 7 (eluted with *n*-hexane/EtOAc 8:2 and 7:3), **6** (1.2 mg), and **11** (7.2 mg) were obtained by CC and HPLC (*n*-hexane/EtOAc 6:4). 

The dried roots of sample 6 (45.2 g) were extracted with EtOH. The extract (838.5 mg) was subjected to silica-gel (20 g) CC using *n*-hexane/EtOAc (gradient) as the eluent to obtain six fractions. From Fr. 4 (eluted with *n*-hexane/EtOAc 9:1), **14** (2.6 mg) was isolated by repeated CC and HPLC (*n*-hexane/EtOAc 8:2). From Fr. 5 (eluted with *n*-hexane/EtOAc 8:2), **9** (5.1 mg) and **3** (1.4 mg) were obtained by CC and HPLC (*n*-hexane/EtOAc 7:3). From Fr. 6 (eluted with EtOAc), **8** (1.4 mg, ratio 7:2), **15** (0.1 mg), and **4** (31.3 mg) were isolated by CC and HPLC (*n*-hexane/EtOAc 3:2, 1:1, and 1:4 for **8**, **15**, and **4**, respectively).

The dried roots of sample 7 (43.5 g) were extracted with EtOH. The extract (1340.9 mg) was subjected to silica-gel (20 g) CC using *n*-hexane/EtOAc (gradient) as the eluent to obtain eight fractions. From Fr. 4 (eluted with *n*-hexane/EtOAc 9:1), **13** (3.3 mg) was isolated by repeated CC and HPLC (*n*-hexane/EtOAc 8:2). From Fr. 5 (eluted with *n*-hexane/EtOAc 8:2), **12** (7.4 mg) was obtained by CC and HPLC (*n*-hexane/EtOAc 8:2).

The dried roots of sample 8 (54.5 g) were extracted with EtOH. The extract (2377.9 mg) was subjected to silica-gel (20 g) CC using *n*-hexane/EtOAc (gradient) as the eluent to obtain eight fractions. From Fr. 4 (eluted with *n*-hexane/EtOAc 9:1), **3** (2.8 mg) was isolated by repeated CC and HPLC (*n*-hexane/EtOAc 8:2). From Fr. 5 (eluted with *n*-hexane/EtOAc 8:2), **8** (1.6 mg; ratio 7:1) was obtained by CC and HPLC (*n*-hexane/EtOAc 7:3). From Fr. 6 (eluted with *n*-hexane/EtOAc 7:3), **8** (4.7 mg) and **4** (196.5 mg) was afforded after purification by CC and HPLC (*n*-hexane/EtOAc 7:3 to 6:4 for **8**, 4:6 to 2:8 for **4**).

The dried roots of sample 9 (*L. limprichtii*) (33.0 g) were extracted with EtOH. The extract (316.6 mg) was subjected to silica-gel (12 g) CC using *n*-hexane/EtOAc (gradient) as the eluent to obtain five fractions. From Fr. 3 (eluted with *n*-hexane/EtOAc 92:8), **3** (1.1 mg) was isolated after purification by HPLC (*n*-hexane/EtOAc 4:1).

### 4.4. (2E)-3-(4-Hydroxy-3-Methoxyphenyl)prop-2-en-1-yl 3-Methylpentanoate *(**2**)*

An oil; [α]_D_^24^ 170 (*c* 0.012, MeOH); IR (neat) 3434 (OH), 1730 (C=O), 1514, 1274 cm^−1^; ^1^H-NMR (CDCl_3_) δ 0.90 (t, *J* = 7.5 Hz, 5′-H_3_), 0.95 (d, *J* = 6.5 Hz, 6′-H_3_), 1.18–1.44 (m, 4′-H_2_), 1.91 (octet, *J* = 6.7 Hz, 3′-H), 2.15 (dd, *J* = 14.8, 8.1 Hz, 2′-H), 2.35 (dd, *J* = 14.8, 5.1 Hz, 2′-H), 3.91 (s, OCH_3_), 4.71 (dd, *J* = 6.6, 1.1 Hz, 1-H_2_), 5.65 (s, OH), 6.14 (dt, *J* = 15.7, 6.6 Hz, 2-H), 6.58 (d, *J* = 15.7 Hz, 3-H), 6.87 (d, *J* = 7.6 Hz, 8-H), 6.90 (dd, *J* = 7.6, 1.5 Hz, 9-H), 6.92 (d, *J* = 1.5 Hz, 5-H); ^13^C-NMR (CDCl_3_) δ 11.3 (C-5′), 19.3 (C-6′), 29.4 (C-4′), 31.9 (C-3′), 41.5 (C-2′), 55.9 (OMe), 65.0 (C-1), 108.3 (C-5), 114.4 (C-8), 120.6 (C-9), 120.9 (C-2), 128.8 (C-4), 134.3 (C-3), 145.8 (C-7), 146.6 (C-6), 173.3 (C-1′); MS (CI) *m*/*z* 278 (M^+^, 100%), 163 (92), 41 (92); HRMS (CI) obs. *m*/*z* 278.1510, calcd for C_16_H_22_O_4_, *M*, 278.1519.

### 4.5. (2Z)- and (2E)- 3-[3,5-Dimethoxy-4-((2E)-3,7-Dimethylocta-2,6-Dien-1-yl)Oxyphenyl]prop-2-Enal *(**8a*** and ***8b**)*

^1^H-NMR (CDCl_3_) δ 1.59 (br s, 9′-H_3_ of **8a**,**b**), 1.66 (br s, 10′-H_3_ of **8a**,**b**), 1.67 (br s, 8′-H_3_ of **8a**,**b**), 1.99-2.11 (m, 4′-H_2_ and 5′-H_2_ of **8a**,**b**), 3.87 (s, OMe of **8b**), 3.90 (s, OMe of **8a**), 4.59 (d, *J* = 7.0 Hz, 1′-H of **8b**), 4.61 (d, *J* = 7.0 Hz, 1′-H of **8a**), 5.03–5.09 (m, 6′-H of **8a**,**b**), 5.52–5.58 (m, 2′-H_2_ of **8a**,**b**), 6.15 (dd, *J* = 11.4, 7.7 Hz, 2-H of **8b**), 6.64 (dd, *J* = 15.7, 7.6 Hz, 2-H of **8a**), 6.60 (s, 5-H and 9-H of **8b**), 6.78 (s, 5-H and 9-H of **8a**), 7.40 (d, *J* = 15.7 Hz, 3-H of **8a**), 7.54 (d, *J* = 11.4 Hz, 3-H of **8b**), 9.68 (d, *J* = 7.6 Hz, 1-H of **8a**), 10.03 (d, *J* = 7.7 Hz, 1-H of **8b**); ^13^C-NMR (CDCl_3_) assigned for **8a**: δ 16.4 (C-10′), 17.6 (C-9′), 25.7 (C-8′), 26.4 (C-5′), 39.6 (C-4′), 56.1 (OMe), 69.6 (C-1′), 105.6 (C-5, 9), 119.8 (C-2′), 123.9 (C-6′), 127.8 (C-2), 129.4 (C-4), 131.7 (C-7′), 139.8 (C-7), 142.0 (C-3′), 152.9 (C-3), 154.0 (C-6, 8), 193.5 (C-1); assigned for **8b**: δ 16.4 (C-10′), 17.6 (C-9′), 25.7 (C-8′), 26.4 (C-5′), 39.6 (C-4′), 56.2 (OMe), 69.6 (C-1′), 107.2 (C-5, 9), 119.9 (C-2′), 123.9 (C-6′), 129.6 (C-4), 129.9 (C-2), 131.7 (C-7′), 138.5 (C-7), 142.0 (C-3′), 148.7 (C-3), 153.7 (C-6, 8), 192.6 (C-1).

## 5. Conclusions

Cacalol (**14**) and its derivative, epicacalone (**15**), were found in the d/k/n complex for the first time, showing that the diversity in the complex is higher than previously known. Two new phenylpropanoids (**2**, **8b**) were also obtained. The basal chemicals in this complex appear to be phenylpropanoids and lupeol, to which sesquiterpenoids are added in some populations. The geographical separation of sesquiterpene-producing populations suggests that the production of this class of compounds evolved independently, perhaps through introgression. *L. limprichtii* was found to produce a phenylpropanoid, showing similarity to the d/k/n complex, which is consistent with DNA data.

## Figures and Tables

**Table 1:** Collection locality and chemical composition of *L. duciformis*, *L. kongkalingensis*, *L. nelumbifolia*, and *L. limprichtii* samples.

Sample No. ^1^	Species	Locality ^2^	Isolated Compounds	Chemotype
1 (2011-12)	*L. duciformis*	Jiajinshan (Baoxing)	**1, 5**	3
2 (2011-29)	*L. nelumbifolia*	Bamei (Daofu)	**3, 7, 9**	3
3 (2011-82)	*L. kongkalingensis*	Yinduo (Xinlong)	**3, 4**	3
4 (2012-10)	*L. kongkalingensis*	Zheduoshan (Kangding)	**9**	4
5 (2012-28)	*L. duciformis*	Yulin (Kangding)	**2, 6, 9, 10, 11**	2
6 (2015-33)	*L. nelumbifolia*	Zhongfengshan (Heishui)	**3, 4, 8, 9, 14, 15**	1
7 (2015-37)	*L. duciformis*	Zhongfengshan (Heishui)	**12, 13**	1
8 (2015-40)	unidentified ^3^	Zhongfengshan (Heishui)	**3, 4, 8**	3
9 (2010-32)	*L. limprichtii*	Gemo (Aba)	**3**	3

^1^ Specimen No. in parenthesis. ^2^ County in parenthesis. ^3^ Resembling *L. kongkalingensis*.

## Supplementary Materials

The following are available online. Table S1: Differences in the sequence of the rDNA ITS1-5.8S-ITS2 region, Figure S1: ^1^H-NMR spectrum of **2**, Figure S2: ^13^C-NMR spectrum of **2**, Figure S3: ^1^H-NMR spectrum of **8**, Figure S4: ^13^C-NMR spectrum of **8**, Figure S5: LCMS profile (total ion chromatogram) of samples 1–6 and 9.
