# Supplementary material for: Terpenoids and Phenylpropanoids in Ligularia duciformis, L. kongkalingensis, L. nelumbifolia, and L. limprichtii
Source: Molecules. 2017 Nov 25;22(12):2062. doi: 10.3390/molecules22122062 (PMC6149998; doi:10.3390/molecules22122062)
Supplement: Supplementary file 1 [file molecules-22-02062-s001.pdf]

Supplementary data

Terpenoids and Phenylpropanoids in *Ligularia duciformis*, *L. kongkalingensis*, *L. nelumbifolia*, and *L. limprichtii*

Chiaki Kuroda [1,*], Ryohei Kobayashi [1], Ayumi Nagata [1], Yumi Nakadozono [1], Taketo Itoh[1], Yasuko Okamoto [2], Motoo Tori [2], Ryo Hanai [3], and Xun Gong [4]

[1] Department of Chemistry, Rikkyo University, Nishi-Ikebukuro, Toshima-ku, Tokyo 171-8501, Japan
[2] Faculty of Pharmaceutical Sciences, Tokushima Bunri University, Yamashiro-cho, Tokushima 770-8514, Japan
[3] Department of Life Science, Rikkyo University, Nishi-Ikebukuro, Toshima-ku, Tokyo 171-8501, Japan
[4] Kunming Institute of Botany, Chinese Academy of Science, Kunming 650201, China

Table S1: Differences in the sequence of the rDNA ITS1-5.8S-ITS2 region.

Figure S1: ^{1}H NMR spectrum of **2**.

Figure S2: ^{13}C NMR spectrum of **2**.

Figure S3: ^{1}H NMR spectrum of **8**.

Figure S4: ^{13}C NMR spectrum of **8**.

Figure S5: LCMS profile (total ion chromatogram) of samples 1-6 and 9.

Table S1. Differences in the sequence of the rDNA ITS1-5.8S-ITS2 region.[1]

Sample no.	ITS1																5.8S	ITS2																		
	8	13	64	65	68	74	108	110	117	132	184	188	204	205	223	224	138	26	27	28	33	41	44	69	70	72	99	101	104	107	151	156	168	199	217	221
1	C	K	G	G	C	C	R	T	T	Y	Y	C	A	S	[6]	[6]	G	T	C	R	G	G	T	Y	C	A	C	C	C	C	Y	S	A	C	Y	C
2	C	T	G	G	C	C	A	T	T	T	C	C	A	C	G	C	G	T	C	G	A	G	T	C	C	A	C	C	C	C	T	G	A	Y	C	C
3	C	T	G	G	C	T	A	T	T	T	C	C	A	C	[6]	[6]	G	[5]	C	G	A	G	T	C	C	A	C	C	Y	C	T	G	A	T	C	Y
4	Y	T	G	G	C	C	A	T	T	T	C	C	A	C	G	C	G	T	C	G	A	G	T	C	Y	A	Y	C	C	Y	Y	G	A	C	C	C
5	C	G	G	G	Y	C	G	T	T	C	T	C	A	C	-	-	G	T	C	A	G	G	T	C	C	A	C	C	C	C	C	G	A	C	T	C
6[2]	C	T	G	G	C	C	A	T	T	T	C	C	A	C	G	C	G	T	C	G	A	G	Y	C	Y	A	C	C	C	C	Y	G	W	Y	C	C
7	C	K	R	K	C	C	R	Y	W	Y	Y	C	R	C	[6]	[6]	G	T	C	R	R	G	T	C	C	A	C	Y	C	C	Y	G	A	C	C	C
8	C	T	G	G	C	C	A	T	T	T	C	Y	A	C	G	C	G	[5]	Y	G	A	G	T	Y	C	A	C	C	C	C	T	G	A	T	C	C
9[3]	C	T	G	G	C	C	A	T	T	T	C	Y	A	C	G	C	G	T	C	R	A	G	T	C	C	A	C	C	C	C	T	G	A	T	C	C
Ref[4]	C	T	G	G	C	C	A	T	T	T	C	C	A	C	G	C	K	T	C	G	G	K	T	Y	C	W	C	C	Y	C	T	G	A	C	C	C

1 K=C+T; R=A+G; S=C+G; W=A+T; Y=C+T; -, deletion.

2 A sequence with A inserted between positions 22 and 23 in ITS2 was also present.

3 Accession no. LC333008 (*L. limprichtii*).

4 A *L. duciformis* sequence in the GenBank/EMBL/DDBJ database (ID=LC128585).

5 A sequence with TT in place of T was also present.

6 Two sequences with and without GC were present.

Figure S1. ^{1}H NMR spectrum of **2**.

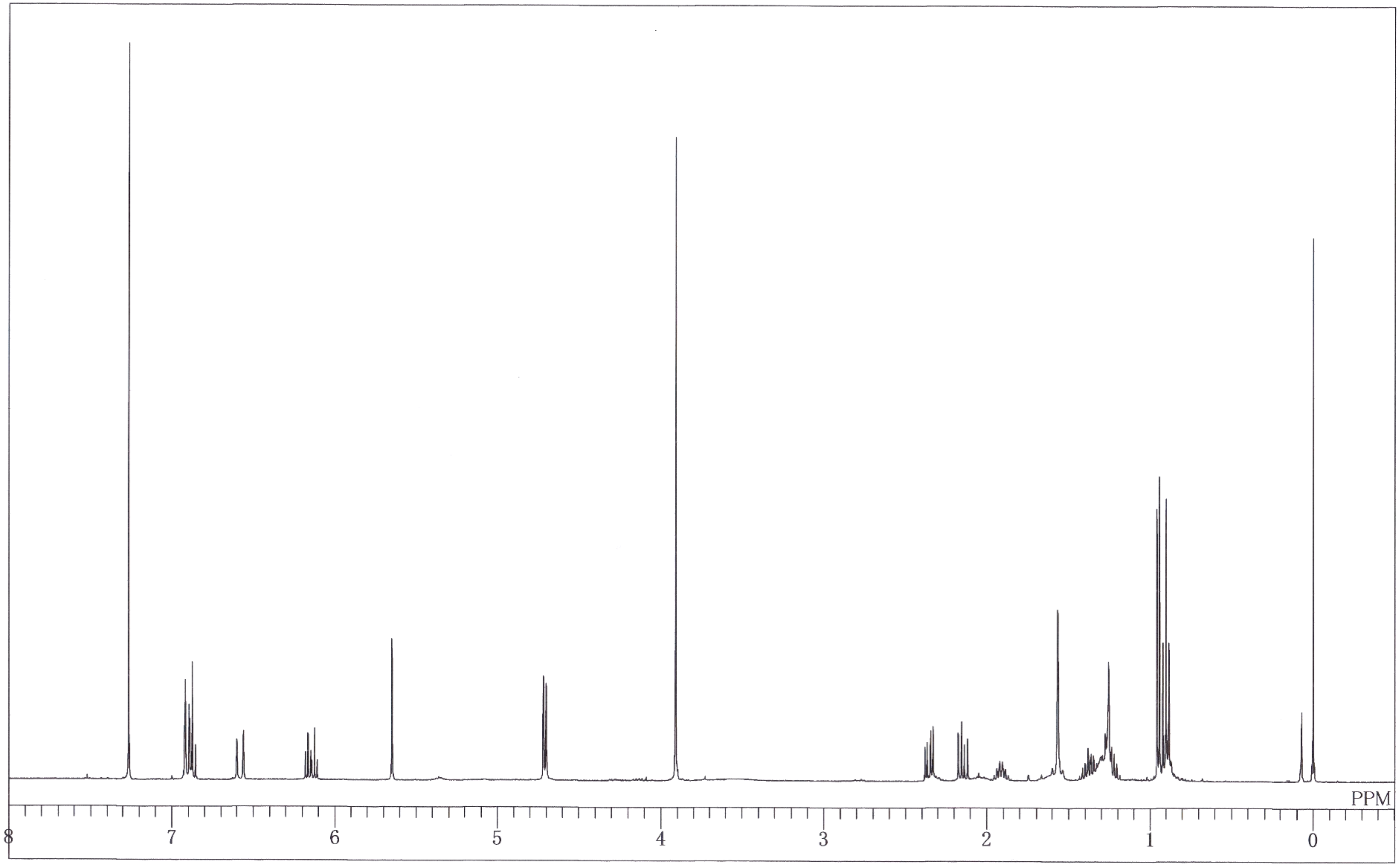

Figure S2. ^{13}C NMR spectrum of **2**.

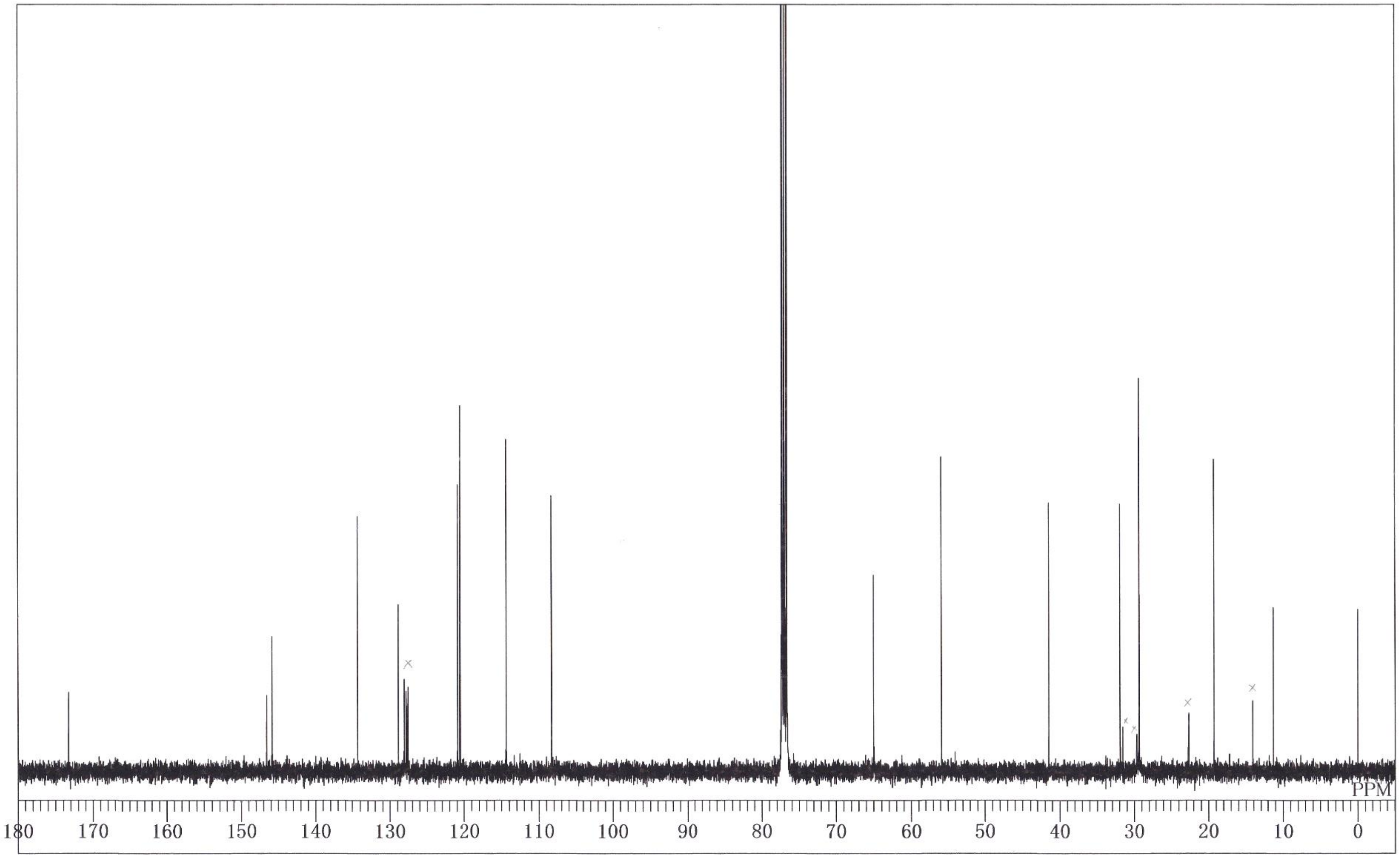

Figure S3. ^{1}H NMR spectrum of **8**.

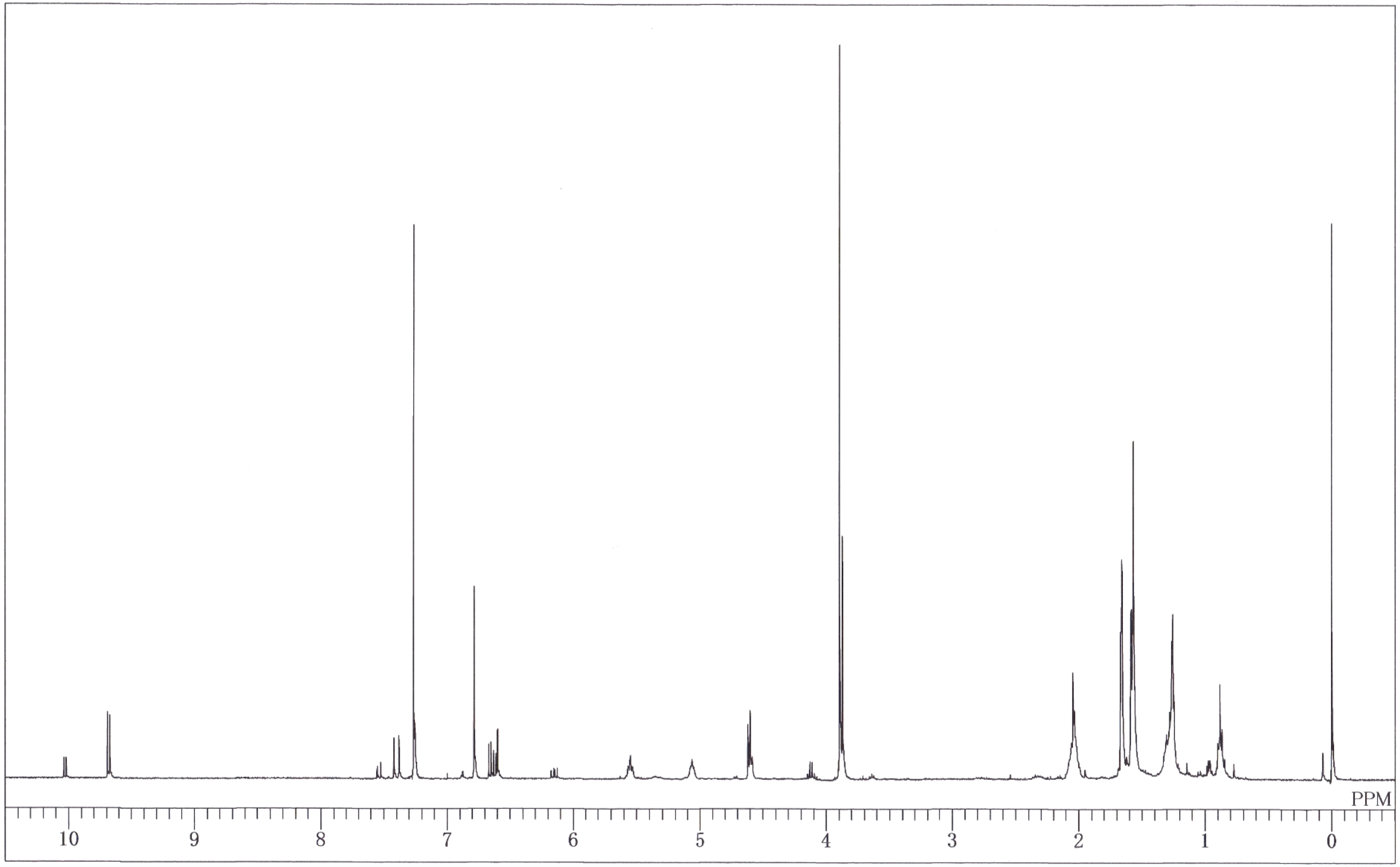

Figure S4. ^{13}C NMR spectrum of **8**.

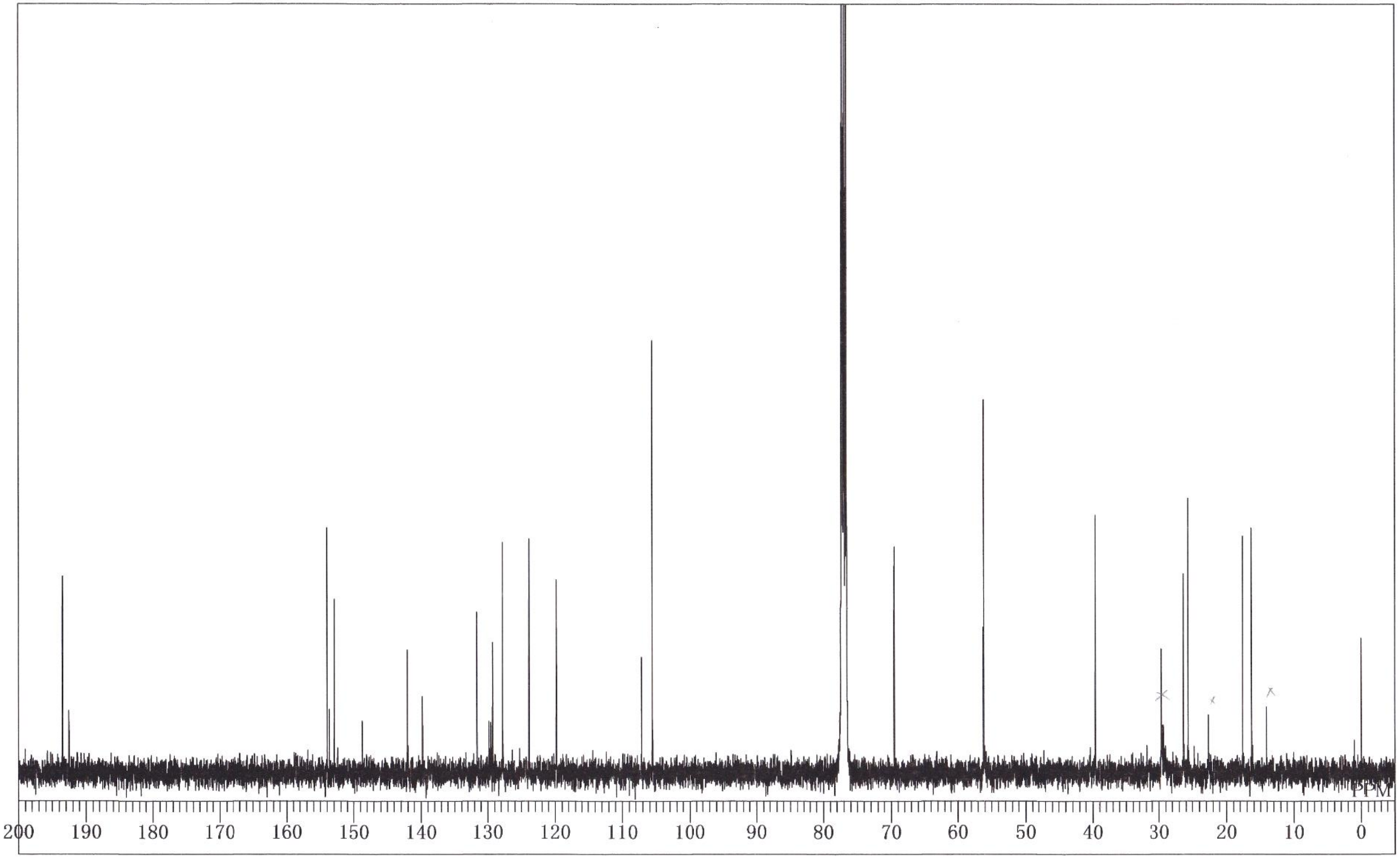

Figure S5. LCMS profile (total ion chromatogram) of samples 1-6 and 9.[1]

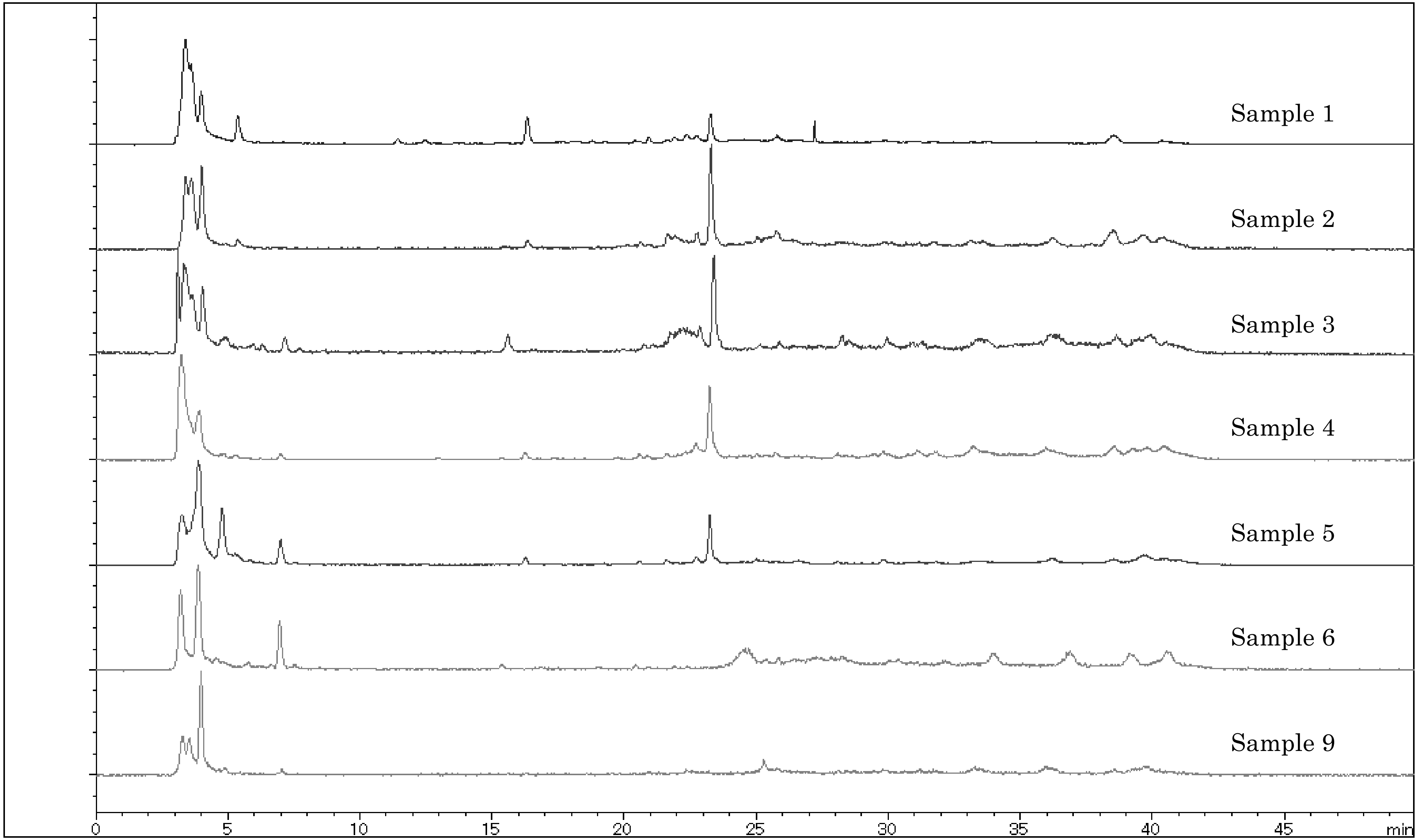

1 A peak at t_R = 23.3 min is an impurity contaminated during sampling procedure.
